# Postural hemodynamic parameters in older persons have a seasonal dependency: A pilot study

**DOI:** 10.1007/s00391-019-01525-3

**Published:** 2019-03-13

**Authors:** Irhad Trozic, Dieter Platzer, Franz Fazekas, Alexander I. Bondarenko, Bianca Brix, Andreas Rössler, Nandu Goswami

**Affiliations:** 1 Gravitational Physiology, Aging and Medicine Research Unit, Physiology Division, Otto Loewi Center of Vascular Biology, Immunity and Inflammation, Medical University of Graz, Neue Stiftingtalstrasse 6, Graz, Austria; 2 Gottfried Schatz Forschungszentrum, Biophysik, Medical University of Graz, Graz, Austria; 3 Department of Neurology, Medical University of Graz, Graz, Austria; 4 Bogomoletz Institute of Physiology of NAS, Kiev, Ukraine; 5 Medical University of Graz, Graz, Austria

**Keywords:** Cardiovascular diseases, Postural blood pressure variability, Orthostatic intolerance, Hemodynamic changes, Meteorological changes, Herz-Kreislauf-Erkrankungen, Posturale Blutdruckvariabilität, Orthostatische Intoleranz, Hämodynamische Veränderungen, Meteorologische Veränderungen

## Abstract

**Aims:**

It is known that blood pressure regulation differs seasonally. It is unknown, however, how the cardiovascular system in patients with a stroke reacts to postural changes in different seasons. The aim was therefore to investigate how different temperatures in cold and warm seasons influence the reactions of haemodynamic mechanisms as well as heart rate variability during a sit-to-stand test in patients with stroke and a control group.

**Methods:**

Hemodynamic responses were assessed in both groups during a sit-to-stand test (5 min sitting followed by 5 min standing) beat to beat within two different seasons. Systolic blood pressure (SBP), diastolic blood pressure (DBP), mean blood pressure (MBP), heart rate (HR), stroke index (SI), cardiac index (CI) and heart rate variability (HRV) were continuously monitored.

**Results:**

During the sitting baseline period delta values of DBP (+15.1 [Standard error (SE) 3.75] mmHg, *p* < 0.05) and MBP (+14.35 [SE 4.18] mmHg, *p* < 0.05) were significantly higher in colder months compared to warmer months whereas SI (−3.86 [SE 1.43] ml/beat/m^2^, *p* < 0.05) and CI (−0.4 [SE 0.11] l/min/m^2^, *p* < 0.05) were lower in colder months compared to warmer months in non-stroke participants. In patients with stroke during sitting, baseline period delta values of DBP (+19.92 [SE 8.03] mmHg, *p* < 0.05) and MBP (+19.29 [SE 8.6] mmHg, *p* < 0.05) were significantly higher in colder months compared to warmer months but SI (−5.43 [SE 1.96] ml/beat/m^2^, *p* < 0.05) was significantly lower in colder months compared to warmer months. After standing, there was a significant decrease in SBP in warmer months (−16.84 [SE 4.38] mmHg, *p* < 0.05) and a decrease in DBP in warmer months (−7.8 [SE 2.3] mmHg, *p* < 0.05) and colder months (−6.73 [SE 1.5] mmHg, *p* < 0.05) in non-stroke participants and a decrease in MBP in warmer months (−12.5 [SE 2.8] mmHg, *p* < 0.05) and colder months (−8.93 [SE 1.8] mmHg, *p* < 0.05) in non-stroke participants and in warmer months (−14.54 [SE 4.1] mmHg, *p* < 0.05) in patients with stroke.

**Conclusion:**

Elderly with and without stroke respond to orthostatic stress with a greater drop in blood pressure in the warmer seasons.

## Zusammenfassung

**Ziele:**

Bekanntermaßen ist die Blutdruckregulation saisonal unterschiedlich ausgeprägt. Unbekannt ist jedoch, wie das Herz-Kreislauf-System bei Patienten mit einem Schlaganfall auf posturale Veränderungen in unterschiedlichen Saisonen reagiert. Ziel war es daher zu untersuchen, wie unterschiedliche Temperaturen in kalten und warmen Saisonen die Reaktionen hämodynamischer Mechanismen sowie die Herzfrequenzvariabilität während eines Sit-to-stand-Tests bei Patienten mit Schlaganfall und einer Kontrollgruppe beeinflussen.

**Methoden:**

Hämodynamische Reaktionen wurden in beiden Gruppen während eines Sit-to-stand-Tests (5 min Sitzen gefolgt von 5 min Stehen) beat to beat innerhalb zweier verschiedener Jahreszeiten untersucht. Der systolische Blutdruck (SBD), der diastolische Blutdruck (DBD), der Puls, der mittlere Blutdruck (MBD), Herzfrequenz (HF), der Schlagindex (SI), Herzindex (HI) sowie diverse Parameter der Herzfrequenzvariabilität (HFV) wurden gemessen.

**Ergebnisse:**

Während der Ruhephase waren in der Kontrollgruppe DBD (+15,1 [Standardfehler (SE) 3,75] mmHg; *p* < 0,05) und MBD (+14,35 [SE 4,18] mmHg; *p* < 0,05) in kälteren Monaten signifikant höher als in wärmeren Monaten, und in kälteren Monaten waren SI (−3,86 [SE 1,43] ml/Schlag/m^2^; *p* < 0,05) und HI (−0,4 [SE 0,11] l/min/m^2^; *p* < 0,05) niedriger als in den wärmeren Jahreszeiten. Bei Patienten mit Schlaganfall waren während der Ruhephase die DBD-Werte (+19,92 [SE 8,03] mmHg; *p* < 0,05) und MBD-Werte (+19,29 [SE 8,6] mmHg; *p* < 0,05) signifikant höher als in den wärmeren Monaten und die SI-Werte (−5,43 [SE 1,96] ml/Schlag/m^2^; *p* < 0,05) im Vergleich zu den wärmeren Monaten in den kälteren Jahreszeiten signifikant erniedrigt. Nach dem Stehen gab es in der Kontrollgruppe einen signifikanten Rückgang des SBD in wärmeren Monaten (−16,84 [SE 4,88] mmHg; *p* < 0,05), einhergehend mit einem Rückgang des DBD sowohl in den wärmeren (−7,8 [SE 2,30] mmHg; *p* < 0,05) als auch den kälteren Monaten (−6,73 [SE 1,5] mmHg; *p* < 0,05). Auch konnte eine Abnahme des MBD in den warmen (−12,5 [SE 2,8] mmHg; *p* < 0,05) und kälteren Monaten (−8,93 [SE 1,8] mmHg; *p* < 0,05) sowohl in der Kontrollgruppe als auch in der Gruppe der Patienten mit Schlaganfall in den wärmeren Monaten (−14,54 [SE 4,1] mmHg; *p* < 0,05) beobachtet werden.

**Schlussfolgerung:**

Ältere Personen mit und ohne Schlaganfall reagieren auf orthostatische Belastung mit einem stärkeren Blutdruckabfall in den wärmeren Jahreszeiten.

## Introduction

It is well known that blood pressure is elevated in winter compared to summer [1, 9]. Specifically, systolic blood pressure decreases with increasing temperature and the exposure temperature has been reported to influence the autonomic nervous system [25]. Orthostasis means upright standing, which induces redistribution of blood to the lower body (by gravitational force) and blood pressure reduction. The reduction in blood pressure during standing is more pronounced in warmer seasons as a result of the effects of higher ambient temperature, hypovolemia (increased sweating, salt and fluid loss), and peripheral vasodilatation [38]. The sympathetic nervous system reacts by increasing heart rate and cardiac contractility thus preventing a collapse of the systemic blood pressure and cerebral perfusion [32].

Aging is characterized by physiological changes that reduce the ability in older people to maintain an upright posture and increase the risk of postural instability, falls, hospitalization and mortality [3, 4, 12–14, 18, 24, 27]. Stroke is a common pathological condition among older people characterized by elevated blood pressure, autonomic dysfunctions, altered baroreflex responses and impaired cerebral autoregulation [8, 28, 39]. Since patients with stroke have a higher incidence of orthostatic hypotension and falls compared to healthy persons, which can worsen the outcome after stroke, it is of importance to acquire more evidence regarding the underlying mechanisms in blood pressure regulation during standing in colder and warmer months [34, 37].

The purpose of this study was to examine how temperatures in two seasons (colder months and warmer months) affect the responses of hemodynamic parameters and heart rate variability during a sit-to-stand test in stroke and non-stroke participants.

## Methods

### Study population

A prospective observational pilot study was carried out at the department of neurology of the Medical University of Graz following the methodological guidelines for pilot studies by Thabane et al. 2010 [35]. The STROBE statement checklist and the EQUATOR guidelines were used to report this observational study [11]. This study included patients 1‑year after stroke (mild stroke) and an age-matched control group without history of stroke. Participants were excluded if they had coexisting comorbidities (referring to the modified Rankin scale >2), neurological illnesses (e.g. epilepsy, dementia and Parkinson’s disease) and intracranial vessel stenosis (Table 1).

**Table 1:** Population characteristics

Characteristics	Stoke group (*n* = 16)	Non-stroke group (*n* = 25)	Sig.
**Population characteristics**			
Male (%)	11 (69)	11 (44)	N. t.
Age (years)	65.81 ± 1.85	63.72 ± 1.39	*p* = 0.371
Height (cm)	173.5 ± 2.22	170.48 ± 1.8	*p* = 0.298
Weight (kg)	83.5 ± 4.09	78.44 ± 3.24	*p* = 0.340
*Antidiabetic medication*			
Biguanide	2 (12.5%)	N. t.	
*Antihypertensive medication*			
ACE inhibitor	6 (37.5%)	1 (4%)	
β1 selective beta blockers	2 (12.5%)	2 (8%)	
Angiotensin II receptor antagonists	1 (6.25%)	2 (8%)	
Thiazide	2 (12.5%)	N. t.	
Calcium channel blockers	2 (12.5%)	N. t.	
**Vascular risk factors**			
Atrial fibrillation	1 (6.25%)	N. t.	
Peripheral arterial disease	2 (12.5%)	N. t.	
Coronary heart disease	2 (12.5%)	N. t.	
Nicotine abuse	2 (12.5%)	1 (4%)	
Obesity	1 (6.25%)	N. t.	
Arterial hypertension	10 (62.5%)	2 (8%)	
Dyslipidemia	8 (50%)	2 (8%)	
Diabetes mellitus	2 (12.5%)	N. t.	
**Stroke characteristics**			
*Stroke etiology*			
Large vessel disease	17%		
Cardioembolic source	42%		
Cryptogenic stroke	42%		
*Initial NIHSS (at hospitalization)*			
Non-stroke symptoms (score: 0)	33%		
Minor stroke (score: 1–4)	67%		
*Baseline NIHSS (at examination)*			
Non-stroke symptoms (score: 0)	58%		
Minor stroke (score: 1–4)	42%		
*Disability (modified Rankin scale)*			
No symptoms after stroke (score: 0)	67%		
No significant disability after stroke (score: 1)	33%		

*N. t.* Not testified

The ethics committee of the Medical University of Graz approved the study. The experimental protocol was performed following the recommendations of the World Medical Association [40]. Informed consent was obtained from all participants in this pilot study following the Declaration of Helsinki. Basic neurological assessment was provided for the participating subjects (clinical neurological examination; extracranial and intracranial duplex sonography) and all subjects received medical clearance to participate prior to performing a single sit-to-stand test.

### Sample size calculation

The selection criteria and the number of participants required to show statistical significance were based on previously published studies regarding hemodynamic responses to orthostatic loading (sit-to-stand test) in patients with stroke [7, 10, 28, 31]. It was estimated that using a sample size calculator with an error probability (a) of 0.05, power (1-β) of 0.80 and an average effect size (d) of 0.5, the number of participants needed to achieve statistical significance was 37.

### Experimental protocol

Initially after arriving in the laboratory the subjects were asked to sit down on a chair. Then following placement of the electrodes, the hemodynamic recording was started. The subjects remained motionless in this position for 5 min (resting phase) and then the participants were asked to stand up. On completion of the 5 min period of standing, the participants were assisted to the seated position. The data were recorded again for 5 min. All reasonable precautions such as assistance in standing and medical personnel availability were in place in case of collapse or syncope.

All investigations were performed between 7:00 a.m. and 11:00 a.m. inside a quiet room maintained at 23–25 °C and 50–55% humidity. Subjects were asked not to eat on the morning of the test and to refrain from consuming coffee and other stimulants (nicotine, thein, etc.) 24 h before the study protocol. This protocol was repeated in two seasons, in cold months (November–April) and in warm months (May–October). In Table 2. the temperature and humidity levels during the 1‑year examination in the City of Graz are summarized (Table 2).

**Table 2:** Temperature and humidity for each month during the assessment in the City of Graz

Months/Parameters	Temperature °C (7 a.m.)	Temperature °C (day)	Humidity % (7 a.m.)	Humidity % (day)
*Colder months*				
Nov 14	6.7	8.1	94	89
Dec 14	0.4	2.2	88	81
Jan 15	0.7	2.5	82	75
Feb 15	0.1	2.2	83	76
Mar 15	2.5	6.3	76	63
Apr 15	7.7	11	65	54
Mean (SE)	3.01 (1.37)	5.38 (1.5)	81.3 (4.09)	73 (5.14)
*Warmer months*				
May 15	13.5	15.7	75	66
Jun 15	17.3	19.7	72	62
Jul 15	20.1	22.7	74	65
Aug 15	18.7	21.9	77	65
Sep 15	13	15.2	80	72
Oct 15	7.9	9.9	91	84
Nov 15	3.5	6.8	88	77
Mean (SE)	13.4 (2.27)	16 (2.26)	79.6 (2.75)	70.14 (3)

### Equipment used and measurements performed

**Fig. 1:**
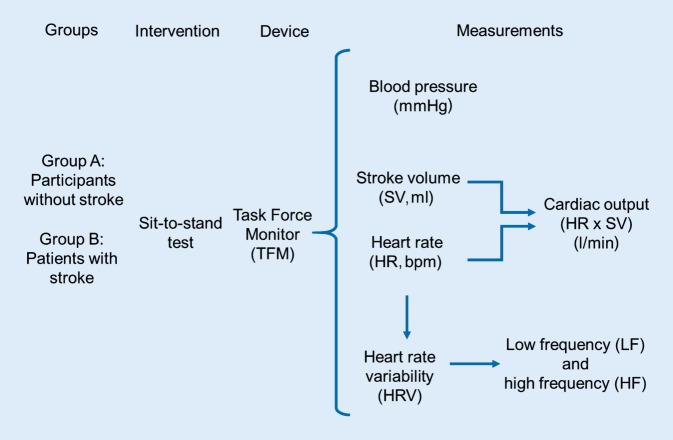
Flow chart showing measured hemodynamic parameters and heart rate variability (HRV) responses via the Task Force Monitor® device

Throughout the study a finger plethysmograph and an upper arm sphygmomanometer were used to measure the arterial blood pressure. After the participant sat down a finger plethysmograph cuff was placed on the middle finger on the right arm at the heart level. The upper arm sphygmomanometer was placed on the left arm. The following hemodynamic and autonomic parameters were measured with a Task Force Monitor® (TFM, CNSystems, Graz, Austria): blood pressure (systolic and diastolic blood pressure), heart rate (bipolar 3‑lead electrocardiograph, ECG), stroke volume (SV), which by definition is the quantity of blood that is ejected into the aorta by each heart beat by the left ventricle and stroke index (SI), which is the stroke volume indexed by the body surface area, cardiac output (CO) which is the volume of circulating blood in 1 min, and cardiac index (CI) which is the cardiac output indexed by the body surface area, power spectral analysis of the heart rate, sympathetic tone characterized by the low frequency of the heart rate variability and the high frequency of the heart rate variability (LF: 0.04–0.15 Hz), where LF (normalized) is the relationship to the low frequency (absolute) and difference between very low frequency and total power, and by this it reduces the influence of very low frequency variations and indicates the sympathetic activity, vagal tone characterized by the high frequency (HF 0.15–0.4 Hz), where HF (normalized) is in relation to the high frequency (absolute) and difference between very low frequency and total power, and by this it reduces the influence of very low frequency variations and indicates the parasympathetic activity, power components of the RR interval (RRI), describe the time interval between two R peaks in the ECG. For details of the methodology related to these parameters, see references ([15, 16, 21, 22]; Fig. 1).

### Statistical analysis

**Fig. 2:**
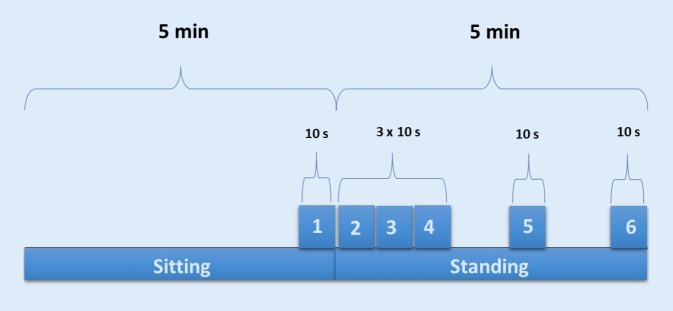
The specific time points (epochs) relevant for the statistical analysis. Epoch 1 (resting phase) = 290–300 sec sitting, epoch 2 = 0–10 sec standing, epoch 3 = 10–20 sec standing, epoch 4 = 20–30 sec standing, epoch 5 = 170–180 sec standing, epoch 6 = 290–300 sec standing

The data analysis was performed using IBM SPSS (version 23; IBM, Armonk, NY, USA) software. Data points (10s) were chosen from distinct phases (sitting, standing) of the protocol (Fig. 2). The time course of the intervention was analyzed with a linear-mixed model (LMM) statistical method that is designed for study with continuous variables (normal distribution), but with not independent residuals and not continuous variances (repeated measures) [5]. Multiple-comparison test with Bonferroni correction was used to analyze the differences between the resting phase (Epoch 1) and epochs 2–6 (Fig. 2). Generalized estimating equations (GEE), a statistical method for longitudinal data with small sample size, was used to analyze: i) seasonal hemodynamic parameters and HRV differences at rest and ii) differences between patients with stroke and non-stroke participants [36]. In both models subjects were specified as a random factor and time course and seasons as two fixed factors.

## Results

Through 2014–2015, a total number of 41 participants (stroke: *n* = 16, non-stroke: *n* = 25) were enrolled. The mean age of patients with stroke was 65.81 ± 1.85 years, and non-stroke participants was 63.72 ± 1.39 years. There were more men in the stroke group (11, 69%). No significant differences in age, height or weight were detected between the two groups (Table 1).

### Hemodynamic and HRV parameters at rest

**Fig. 3:**
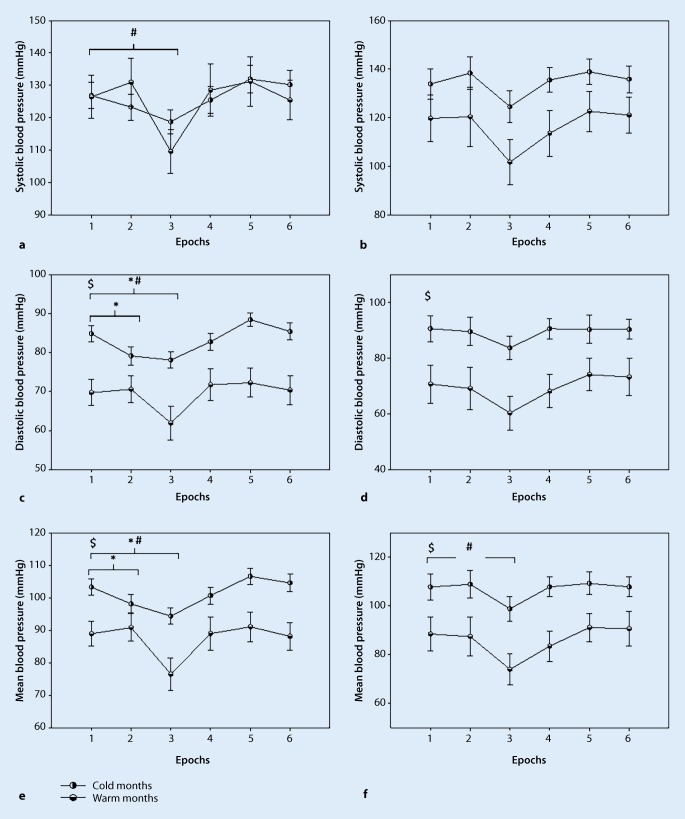
Systolic (**a**,**b**), diastolic (**c**,**d**) and mean blood pressure (**e**,**f**) in patients with stroke and non-stroke participants (mean ± SE), (*$* significant seasonal differences at rest, * significant time interaction between the resting phase and epoch 2–6. *#* significant time interaction between the resting phase (epoch 1) and epochs 2–6 in warmer months, *p* ≤ 0.05 significance) **a**, **c** and **e** non-stroke participants; **b**, **d** and **f** stroke patients

**Fig. 4:**
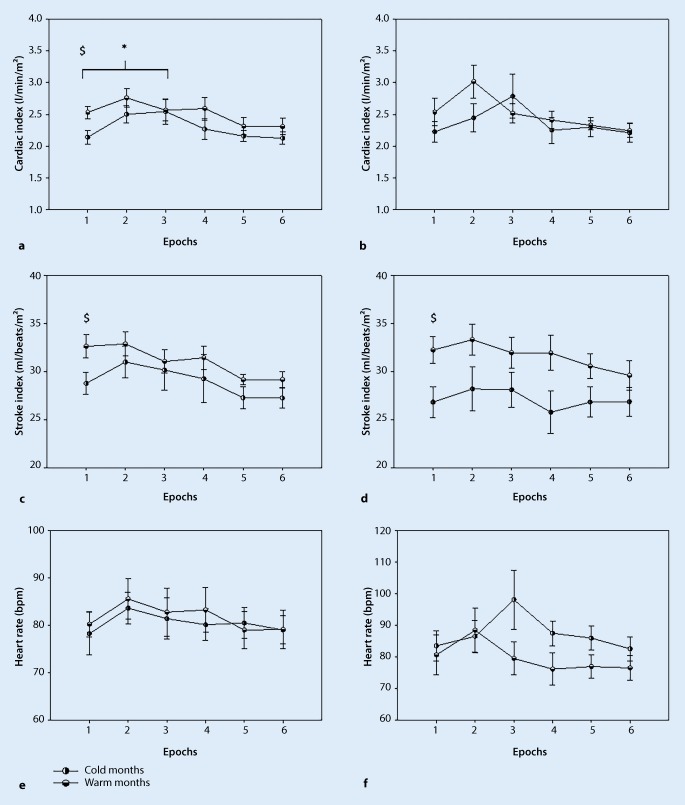
Cardiac index, stroke index and heart rate in patients with stroke and non-stroke participants (mean ± SE), (*$* significant seasonal differences at rest, * significant time interaction between the resting phase (epoch 1) and 2–6 epoch in colder months, *#* significant time interaction between the resting phase (epoch 1) and 2–6 epoch in warmer months, *p* ≤ 0.05 significance) **a**, **c** and **e** non-stroke participants; **b**, **d** and **f** stroke patients

During the resting phase in non-stroke participants, the systolic blood pressure remained stable in both seasons with no significant changes (warm months: 126.439 ± 6.311, cold months: 126.864 ± 3.90, Fig. 3a), the diastolic blood pressure was significantly higher in cold months compared to warm months (warm months: 69.731 ± 3.202, cold months: 84.846 ± 2.018, *p* < 0.05, Fig. 3c), and the mean blood pressure was significantly higher in cold months compared to warm months (warm months: 88.962 ± 3.6422, cold months: 103.318 ± 4.1825, *p* < 0.05, Fig. 3e). Furthermore, the cardiac index was significantly lower in cold months compared to warm months (warm months: 2.533 ± 0.0945, cold months: 2.142 ± 0.1052, *p* < 0.05, Fig. 4a) and the stroke index was significantly lower in cold months compared to warm months (warm months: 32.641 ± 1.1510, cold months: 28.781 ± 1.4382, *p* < 0.05, Fig. 4c). The heart rate, RR-interval, high frequency (normalized) and low frequency (normalized) remained stable in both seasons with no significant changes.

During the resting phase, in patients with stroke, the systolic blood pressure remained stable in both seasons with no significant changes (Fig. 3b), the diastolic blood pressure was significantly higher in cold months compared to warm months (warm months: 70.704 ± 6.380, cold months: 90.624 ± 4.522, *p* < 0.05, Fig. 3d) and the mean blood pressure was significantly higher in cold months compared to warm months (warm months: 88.505 ± 6.499, cold months: 107.803 ± 5.084, *p* < 0.05, Fig. 3f). The stroke index was significantly lower in cold months compared to warm months (warm months: 32.258 ± 1.289, cold months: 26.820 ± 1.552, *p* < 0.05, Fig. 4d). The cardiac index, heart rate, RR interval, high frequency (normalized) and low frequency (normalized) values remained unchanged in both seasons with no significant changes.

### Postural hemodynamic and HRV parameters responses in two seasons

In this section the hemodynamic and HRV responses to orthostatic loading (standing) compared to the resting phase are shown. In the non-stroke participants in the warm months, the systolic blood pressure decreased significantly during 20 sec of standing compared to the resting phase (Epoch 1: 126.439 ± 7.295, Epoch 3: 109.590 ± 7.295, *p* < 0.05, Fig. 3a). In cold months, the diastolic blood pressure decreased significantly after the first 10 sec of standing (Epoch 1: 84.847 ± 1.987, Epoch 2: 79.151 ± 1.987, *p* < 0.05, Fig. 3c), and decreased significantly after 20 sec of standing compared to the resting phase (Epoch 1: 84.847 ± 1.987, Epoch 3: 78.113 ± 1.987, *p* < 0.05, Fig. 3c). In the warm months, the diastolic blood pressure decreased significantly after the 20 sec of standing compare to the resting phase (Epoch 1: 69.731 ± 3.804, Epoch 3: 61.942 ± 3.804, *p* < 0.05, Fig. 3c). In the cold months, the mean blood pressure decreased significantly after 10 sec of standing (Epoch 1: 103.318 ± 2.454, Epoch 2: 98.139 ± 2.454, *p* < 0.05, Fig. 3e), and again after 20 sec of standing as compared to the resting phase (Epoch 1: 103.318 ± 2.454, Epoch 3: 94.385 ± 2.454, *p* < 0.05, Fig. 3e). In the warm months, the mean blood pressure decreased significantly after the 20 sec of standing compared to the resting phase (Epoch 1: 88.962 ± 4.585, Epoch 3: 76.492 ± 4.585, *p* < 0.05, Fig. 3e). In cold months, the cardiac index increased significantly after 20 sec of standing compared to the resting phase (Epoch 1: 2.120 ± 0.128, Epoch 3: 2.525 ± 0.128, *p* < 0.05, Fig. 4a). The stroke index, heart rate, RR interval, low frequency (normalized) and high frequency (normalized) values remained stable to the end of the sit-to-stand protocol in both seasons.

**Fig. 5:**
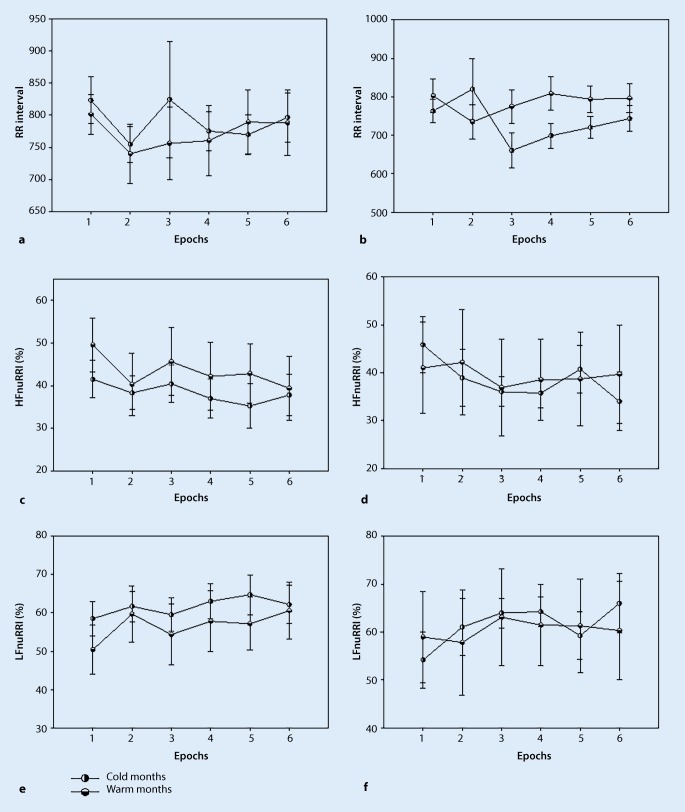
RR interval, high frequency (normalized) and low frequency (normalized) in patients with stroke and non-stroke participants (values are mean ± SE) **a**, **c** and **e** non-stroke participants; **b**, **d** and **f** stroke patients

In patients with stroke, the mean blood pressure decreased significantly in the warm months during 20 sec of standing compared to the resting phase (Epoch 1: 88.505 ± 6.397, Epoch 3: 73.958 ± 6.397, *p* < 0.05, Fig. 3f). The systolic blood pressure and the diastolic blood pressure remained stable to the end of the sit-to-stand protocol in both seasons. Also, cardiac index, stroke index, heart rate, RR interval, low frequency (normalized) and high frequency (normalized) remained stable to the end of the sit-to-stand protocol in both seasons (Fig. 5; Tables 3 and 4).

**Table 3:** Time course of hemodynamic parameters and heart rate variability (HRV) responses in colder months

		Epochs						Sig.
Parameters	Groups	Epoch 1	Epoch 2	Epoch 3	Epoch 4	Epoch 5	Epoch 6	
SBP [mmHg]	Control	126.9 (4.0)	123.3 (3.98)	118.77 (3.98)*	125.5 (3.97)	129.6(4.02)	128.1 (4.02)	*p* < 0.05
	Stroke	133.9 (5.73)	138.4 (5.73)	124.56 (5.73)	135.53 (5.73)	138.9 (5.73)	135.87 (5.73)	
DBP [mmHg]	Control	84.84 (1.98)	79.15 (1.98)*	78.11 (1.98)*	82.8 (1.98)	87.77 (2.02)	85.29 (2.02)	*p* < 0.05
	Stroke	90.62 (4.29)	89.55 (4.29)	83.7 (4.29)	90.6 (4.29)	90.32 (4.29)	90.34 (4.29)	
MBP [mmHg]	Control	103.31 (2.45)	98.13 (2.45)	94.4 (2.45)*	100.71 (2.45)	105.95 (2.49)	103.8 (2.49)	*p* < 0.05
	Stroke	107.80 (4.71)	108.86 (4.71)	98.82 (4.71)	107.81 (4.71)	109.26 (4.71)	107.84 (4.71)	
SI [ml/beat/m^2^]	Control	28.64 (1.61)	30.87 (1.61)	30.03 (1.61)	29.12 (1.61)	27.14 (1.61)	27.13 (1.60)	–
	Stroke	26.82 (2.15)	28.34 (2.19)	28.27 (2.19)	26.57 (2.30)	26.98 (2.19)	27.01 (2.19)	
CI [l/min/m^2^]	Control	2.12 (0.12)	2.5 (0.12)	2.52 (0.12)*	2.24 (0.12)	2.13 (0.12)	2.13 (0.12)	*p* < 0.05
	Stroke	2.22 (0.21)	2.43 (0.22)	2.77 (0.22	2.30 (0.23)	2.28 (0.22)	2.19 (0.22)	
HR [bpm]	Control	78.20 (3.38)	83.57 (3.38)	81.4 (3.38)	80.09 (3.38)	80.87 (3.42)	78.98 (3.38)	–
	Stroke	83.43 (4.47)	86.26 (4.64)	97.84 (4.64)	87.72 (5.04)	85.71 (4.64)	82.27 (4.64)	
RRI [ms]	Control	823.54 (42.75)	754.50 (42.75)	824.68 (42.75)	775.18 (42.75)	782.32 (43.43)	796.8 (42.75)	–
	Stroke	763.07 (48.99)	836.57 (50.66)	676.9 (50.66)	726.15 (54.69)	737.09 (50.66)	759.95 (50.66)	
LFnuRRI [%]	Control	58.48 (4.70)	58.70 (4.83)	58.4 (4.75)	61.86 (4.75)	63.60 (4.75)	62.19 (4.70)	–
	Stroke	54.17 (5.95)	58.55 (6.18)	58.12 (6.33)	58.4 (6.33)	56.66 (6.06)	63.41 (6.06)	
HRnuRRI [%]	Control	41.51 (4.70)	41.3 (4.83)	41.6 (4.75)	38.13 (4.75)	36.4 (4.75)	37.8 (4.70)	–
	Stroke	45.82 (5.95)	41.45 (6.18)	41.87 (6.33)	41.6 (6.33)	43.33 (6.06)	36.6 (6.06)	

* significant time interaction between baseline and epochs 2–6 in colder months. Values are mean and (±SE)

**Table 4:** Time course of hemodynamic parameters and heart rate variability (HRV) responses in warmer months

		Epochs						Sig.
Parameters	Groups	Epoch 1	Epoch 2	Epoch 3	Epoch 4	Epoch 5	Epoch 6	
SBP [mmHg]	Control	126.44 (7.29)	130.94 (7.29)	109.6 (7.29)*	128.51 (7.29)	130.14 (7.36)	124.37 (7.36)	*p* < 0.05
	Stroke	119.8 (8.60)	120.43 (8.60)	101.74 (8.60)	113.60 (8.60)	122.66 (8.60)	121.11 (8.60)	
DBP [mmHg]	Control	69.73 (3.80)	70.58 (3.80)	61.94 (3.80)*	71.76 (3.80)	72.81 (3.84)	70.92 (3.84)	*p* < 0.05
	Stroke	70.70 (6.42)	69.11 (6.42)	60.31 (6.42)	68.12 (6.42)	74.12 (6.42)	73.23 (6.42)	
MBP [mmHg]	Control	88.96 (4.58)	90.84 (4.58)	76.5 (4.58)*	88.99 (4.58)	91.31 (4.63)	88.37 (4.63)	*p* < 0.05
	Stroke	88.5 (6.39)	87.45 (6.39)	73.95 (6.39)*	83.5 (6.39)	91.15 (6.39)	90.66 (6.39)	*p* < 0.05
SI [ml/beat/m^2^]	Control	32.40 (1.14)	32.96 (1.16)	32.13 (1.16)	31.53 (1.16)	29.47 (1.14)	29.48 (1.14)	–
	Stroke	32.25 (1.52)	33.33 (1.52)	31.97 (1.52)	31.94 (1.52)	30.58 (1.52)	29.6 (1.52)	
CI [l/min/m^2^]	Control	2.52 (0.13)	2.75 (0.13)	2.55 (0.13)	2.58 (0.13)	2.31 (0.13)	2.3 (0.13)	–
	Stroke	2.53 (0.18)	3.01 (0.18)	2.51 (0.18)	2.41 (0.18)	2.33 (0.18)	2.24 (0.18)	
HR [bpm]	Control	79.81 (3.90)	84.43 (4.03)	81.64 (4.03)	82.1 (4.03)	78.33 (3.92)	78.46 (3.92)	–
	Stroke	80.58 (5.23)	88.37 (5.23)	79.42 (5.23)	76.09 (5.23)	76.9 (5.23)	76.46 (5.23)	
RRI [ms]	Control	819.28 (57.21)	764.99 (58.97)	781.26 (58.97)	785.41 (58.97)	794.29 (57.41)	793.06 (57.41)	–
	Stroke	802.61 (41.88)	734.7 (41.88)	774.61 (41.88)	808.52 (41.88)	793.61 (41.88)	796.38 (41.88)	
LFnuRRI [%]	Control	47.92 (6.54)	51.4 (6.95)	51.7 (6.68)	55.15 (6.68)	57.23 (6.57)	60.65 (6.57)	–
	Stroke	58.97 (10.13)	56.29 (10.35)	60.23 (10.35)	61.45 (10.13)	61.31 (10.13)	60.29 (10.13)	
HRnuRRI [%]	Control	52.07 (6.54)	48.59 (6.95)	48.29 (6.68)	44.84 (6.68)	42.76 (6.57)	39.34 (6.57)	–
	Stroke	41.02 (10.13)	43.7 (10.35)	39.76 (10.35)	38.54 (10.13)	38.68 (10.13)	39.7 (10.13)	

* significant time interaction between baseline and 2–6 epoch in warmer months. Values are mean and (±SE)

## Discussion

This study analyzed seasonal variations in postural blood pressure changes and differences in postural blood pressure change between stroke and non-stroke participants. The main findings of the study were as follows: 1) at rest diastolic blood pressure and mean blood pressure were higher and stroke index was lower in both groups in colder months, 2) resting cardiac index was lower in non-stroke participants in colder months and 3) standing (10–30 sec) resulted in:

- Decrease in systolic blood pressure in warmer months in non-stroke participants.
- Decrease in diastolic blood pressure and mean blood pressure in both seasons in non-stroke participants.
- Decrease in mean blood pressure in warmer months in patients with stroke.
- Increase in cardiac index in colder months in non-stroke participants.
- No significant differences at rest or during the sit-to-stand test for hemodynamic parameters and heart rate variability were detected between patients with stroke and non-stroke participants in both seasons.

Earlier studies reported the effects of seasons on the cardiovascular system in healthy participants. Sega et al. [33] and Brennan et al. reported that blood pressure was lower in summer and higher in winter and heart rate was similar over the four seasons [6]. Radak and Tanaskovic explained the seasonal differences in blood pressure by the effect of heat on blood pressure regulation (vasodilation) and dehydration in summer and by an increased sympathetic tone in winter [30]. This is supported by the present findings with a higher resting diastolic blood pressure and resting mean blood pressure in colder months compared to warmer months in both groups. The resting heart rate was similar in both seasons, but the resting stroke index was lower in colder months in both groups and resting cardiac index was lower in colder months but only in non-stroke participants.

It is interesting that in this study the resting systolic blood pressure remained unchanged and the resting diastolic and resting mean blood pressure showed a reduction in warmer months. We postulate that this could have arisen due to the constant indoor conditions of the room where the testing sessions were carried out. A previously published work reported that indoor temperature mostly contributes to the variability in systolic blood pressure. Also, systolic blood pressure variability is more frequent in warmer countries than in countries with colder climate [2]. Furthermore, antihypertensive medications are mostly used in the case of elevated diastolic blood pressure and less frequently in the case of elevated systolic blood pressure [26]. The intake of antihypertensive medications can lead to a significant reduction in blood pressure and orthostatic hypotension in summer [17]. Some of patients in this study were on medications (specifically, antihypertensive medication) and this could have led to decreases in blood pressure; however, orthostatic hypotension was not observed in warmer months in the patients, which is in contrast to the results of Huang et al. [17]. We speculate that the stable resting systolic blood pressure and the reduced resting diastolic and resting mean blood pressures in the warmer months could be due to the stable indoor temperature in the room where the tests were carried out, the colder Austrian climate, and/or due to the intake of antihypertensive medication in older persons as well as in patients with stroke.

There were two earlier studies that analyzed how blood pressure during standing is influenced by different seasons. Radak and Tanaskovic and Weiss et al. noted that blood pressure drop during standing was higher in summer than in winter, and that the incidence of orthostatic hypotension was higher in summer (64%) than in winter [30, 38]. Additionally, Weiss et al. reported a faster heart rate during standing in winter which they associated with increased sympathetic activity [38]. These earlier findings are comparable to the present results with a decline in systolic blood pressure after 10–30 sec of standing in warmer months, diastolic blood pressure and mean blood pressure after 10–30 sec of standing in both seasons but the decrease was more pronounced in warm months in non-stroke participants. Also, in patients with stroke there was a significant decline in mean blood pressure but only in warmer months. The result differs from the earlier observation in the heart rate, where there was no increase during standing up in both seasons. Due to the simple orthostatic challenge protocol (sit-to-stand test) and the stable indoor temperature in the laboratory, there were no cases of orthostatic hypotension in our study. As an individual stands up, blood moves to the lower body due the gravitational force and this could potentially lead to reductions in blood pressure. It is well known that the decrease in blood pressure during standing is more pronounced in warmer season. Higher ambient temperature is associated with increased sweating, salt and fluid loss, hypovolemia and peripheral vasodilatation [38]. The sympathetic nervous system acts to maintain the blood pressure by increasing heart rate and cardiac contractility and reducing blood in non-cutaneous areas. This prevents a collapse of the systemic blood pressure and cerebral perfusion [32].

Overall, in patients 1 year after stroke and older participants without stroke, greater postural blood pressure decreases in warmer months compared to colder months were observed: however, there were no cases of orthostatic hypotension or syncope in warmer months. In the patient’s group, one had a minor stroke at hospitalization and overall no disability after stroke. Previously it has been reported that orthostatic hypotension is common in patients with pre-stroke morbid condition, those with severe stroke and/or post-stroke disability and prolonged immobilization [19, 20, 23, 29]. As patients in this study have a mild stroke it is possible that, their cardiovascular and autonomic mechanisms were able to respond effectively to orthostatic loading (sit-to-stand test). We speculated that in patients with severe stroke and disability and those with prolonged immobilization, orthostatic hypotension and syncope would occur. These results emphasize the importance of further studies with severe stroke and post-stroke patients.

The study has several limitations. Firstly, the smaller number of enrolled participants in this study compared to similar studies that investigated seasonal variation in blood pressure and orthostatic tolerance is a major limitation. Nevertheless, as studies which examine patients with stroke across seasons are rare, this pilot study can be used as a basis for calculating sample size in further studies. Secondly, there are differences in the composition of the groups, with a larger number of males enrolled in the stroke group due the fact that the incidence of stroke is higher in men compared to women. However, as our analysis focused between stroke and non-stroke groups, this is not seen as a major limitation.

In conclusion, our study showed that resting blood pressure values depend on the season, being higher in colder months in both groups. Furthermore, standing up leads to a significant decrease in systolic blood pressure in warmer months, significant decrease in diastolic blood pressure in both seasons in non-stroke participants and significant decrease in mean blood pressure in warmer months in both groups. The study showed that patients with stroke and older persons without stroke respond to orthostatic loading in a comparable manner with greater decrease in postural blood pressure in warmer months. Furthermore, patients 1 year after stroke (minor stroke) and without any disabilities showed none of the signs of orthostatic hypotension despite postural blood pressure reduction in warmer months.

The authors suggest the need for further studies with patients with severe stroke and post-stroke disabilities. To our knowledge this is the first study that has examined the seasonal differences of hemodynamic parameters and heart rate variability during a sit-to-stand test in patients with stroke.
